# How to monitor cardiovascular function in critical illness in resource-limited settings

**DOI:** 10.1097/MCC.0000000000000830

**Published:** 2021-04-08

**Authors:** Chaisith Sivakorn, Marcus J. Schultz, Arjen M. Dondorp

**Affiliations:** a Department of Clinical Tropical Medicine; b Mahidol–Oxford Tropical Medicine Research Unit, Faculty of Tropical Medicine Mahidol University, Bangkok, Thailand; c Department of Intensive Care & Laboratory of Experimental Intensive Care and Anesthesiology (L.E.I.C.A.), Academic Medical Center, University of Amsterdam, Amsterdam, The Netherlands; d Centre for Tropical Medicine and Global Health, Nuffield Department of Medicine, Oxford University, Oxford, UK

**Keywords:** cardiovascular failure, cardiovascular monitoring, hemodynamic failure, hemodynamic monitoring, low-income and middle-income countries, resource-limited settings, shock

## Abstract

**Purpose of review:**

Hemodynamic monitoring is an essential component in the care for critically ill patients. A range of tools are available and new approaches have been developed. This review summarizes their availability, affordability and feasibility for hospital settings in resource-limited settings.

**Recent findings:**

Evidence for the performance of specific hemodynamic monitoring tools or strategies in low-income and middle-income countries (LMICs) is limited. Repeated physical examination and basic observations remain a cornerstone for patient monitoring and have a high sensitivity for detecting organ hypoperfusion, but with a low specificity. Additional feasible approaches for hemodynamic monitoring in LMICs include: for tissue perfusion monitoring: urine output, skin mottling score, capillary refill time, skin temperature gradients, and blood lactate measurements; for cardiovascular monitoring: echocardiography and noninvasive or minimally invasive cardiac output measurements; and for fluid status monitoring: inferior vena cava distensibility index, mini-fluid challenge test, passive leg raising test, end-expiratory occlusion test and lung ultrasound. Tools with currently limited applicability in LMICs include microcirculatory monitoring devices and pulmonary artery catheterization, because of costs and limited added value. Especially ultrasound is a promising and affordable monitoring device for LMICs, and is increasingly available.

**Summary:**

A set of basic tools and approaches is available for adequate hemodynamic monitoring in resource-limited settings. Future research should focus on the development and trialing of robust and context-appropriate monitoring technologies.

## INTRODUCTION

The level of patient monitoring and organ support feasible in the care for patients with critical illness differs substantially around the globe, and is mainly driven by availability and affordability of the different tools. Hemodynamic monitoring is an essential part of critical care as many patients in the ICU experience hemodynamic instability for a large variety of reasons. 

**Box 1:**
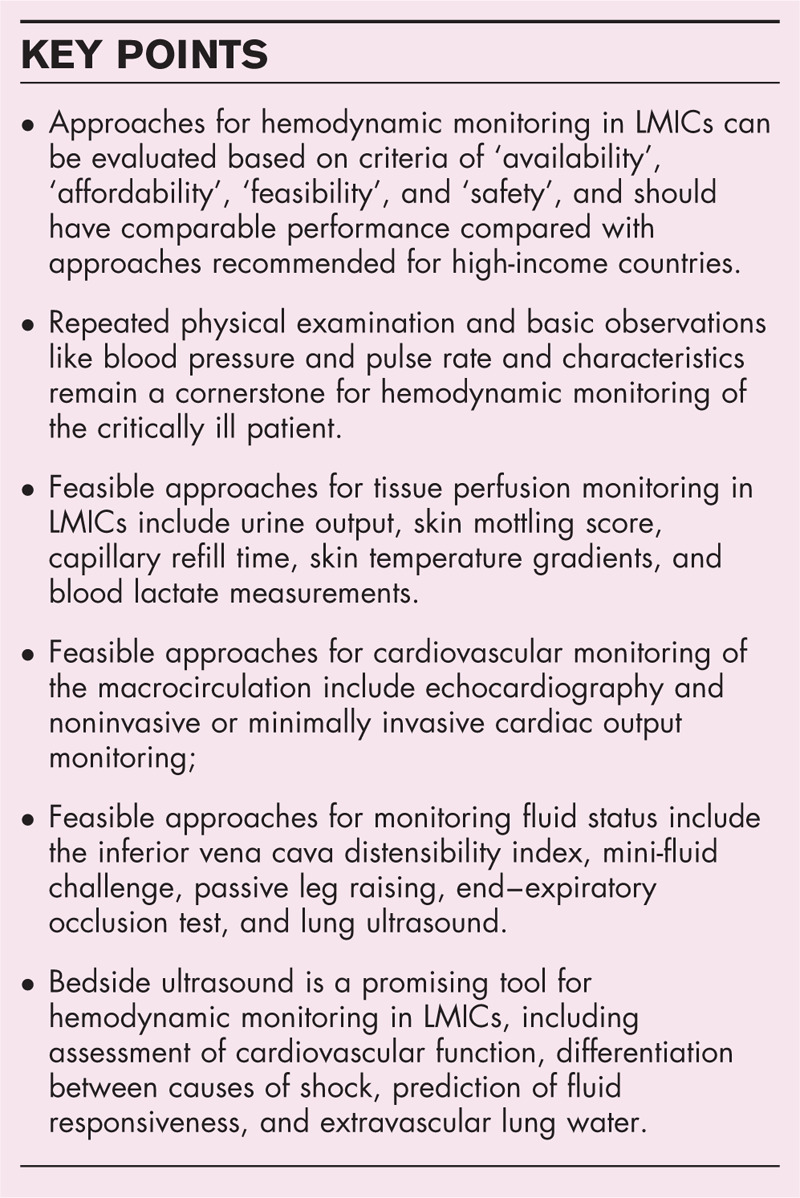
no caption available

Shock is defined as insufficient perfusion at the tissue level, and the ultimate hemodynamic parameter to monitor is perfusion of the microcirculation of vital organs. Yet, quantifying microcirculatory flow is challenging. Clinical assessment of for instance nail bed or skin perfusion or acute changes in organ function of the brain, kidney or other organs, provide an indication for the adequacy of tissue perfusion. There are techniques to quantify local tissue perfusion, and some laboratory parameters, such as blood lactate, provide a rough estimate for overall tissue perfusion.

In clinical practice, however, we rely mainly on monitoring macrocirculatory parameters including cardiac function, intravascular filling status, blood pressure and, derived from these, vascular resistance. These will guide clinical management, including fluid therapy, vasopressor and inotropic drugs, and other interventions aiming to optimize cardiac output, yet avoiding fluid overload causing tissue and pulmonary edema. Hemodynamic monitoring of the macrocirculation include simple clinical assessments, non-invasive and invasive monitoring tools.

The optimal approach and choice of devices and approaches are different in settings with limited resources, and will be guided by costs, complexity of maintenance, human and material infrastructure, safety, and other factors. We evaluated the value of clinical parameters and monitoring devices in LMICs, based on criteria of availability, affordability, and feasibility [1^▪▪^].

## SEARCH STRATEGIES

A literature search was performed using the Medline OVID, EMBASE Cochrane, and PubMed databases. Our search was restricted to studies in adult patients but not to specific years of publication, although with an emphasis on manuscripts published over the last 5 years. The following search terms were used: ‘cardiovascular monitoring’ OR ‘hemodynamic monitoring’ OR ‘invasive’ OR ‘non-invasive’ OR ‘device’ OR ‘critical illness’ OR ‘critically ill’ OR ‘LMICs’. Furthermore, subject terms were also combined with terms referring to specific monitoring techniques, such as ‘ultrasound’ OR ‘echocardiogram’. Reference lists of identified manuscripts were hand-searched to identify further relevant publications.

## TISSUE PERFUSION MONITORING

### Clinical assessment

Simple clinical assessments can provide crucial information on the adequateness of the microcirculation of vital organs, and other tissues. Acute changes in consciousness, anxiety, confusion, or delirium can all be a sign of decreased brain perfusion. Oligo or anuria can be a sign of decreased renal perfusion. Deep breathing or tachypnea can be the respiratory compensation for a lactic acidosis that may originate from anaerobic glycolysis because of inadequate tissue perfusion. Bowel dysfunction can be caused by decreased gut perfusion. Cold extremities, including the tip of the nose, fingers, legs, and toes, as well as mottling of the skin can denote shock. In general, the specificity of these clinical parameters is limited, as these can all have alternative causes. However, their assessments do not require resources apart from skilled medical staff, and it is important to monitor these parameters in the critically ill patients frequently. Some of these parameters are reviewed more systematically below.

### Urine output

In general, an adequate urine output of more than 0.3--0.5 ml/kg/h [2] is considered an indicator of adequate renal blood flow [3], and thus of an adequate intravascular filling status, assuming normal renal tubular function. Urine output has also been evaluated as a resuscitation endpoint in patients with septic shock. In a multicentric observational trial, the presence of persistent oliguria during the ICU stay is associated with higher ICU and hospital mortality [4].

### Skin mottling score

**FIGURE 1:**
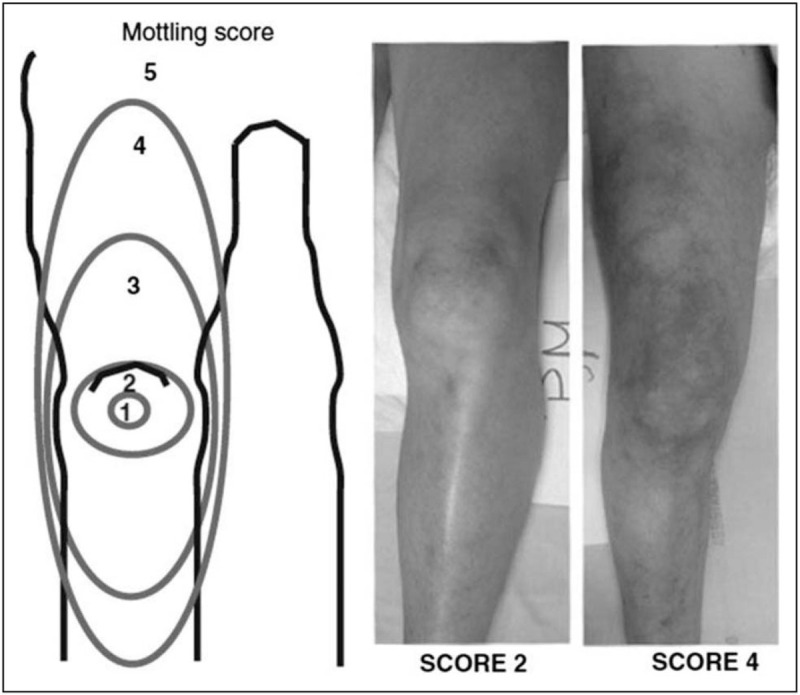
Skin mottling score. Reproduced with permission from Ait-Oufella *et al.*[6].

Skin mottling reflects microcirculatory alterations in the skin [5]. Patchy skin discolorations occur because of heterogenic small vessel vasoconstriction that usually start around the knees and elbows in patients with shock. The skin mottling score is easy to assess at the bedside, using a scale from 0 (‘no mottling’) to 5 (‘grave mottling’) (Table 1 and Fig. 1), and correlates with blood lactate concentrations, urine output, degree of organ dysfunction, and in-hospital case fatality in patients with sepsis or septic shock [6]. Patients whose mottling score improved during the resuscitation period showed better survival. The prognostic value of the skin mottling score was confirmed in several cohort studies of critically ill patients [7,8], and had good reproducibility and small interobserver variability. However, assessing skin mottling is difficult in patients with a dark skin color [8].

**Table 1:** Skin mottling score

Score		Description
0	No	No mottling
1	Modest	Coin size, localized to the center of the knee
2	Moderate	Mottling does not exceed the superior edge of the kneecap
3	Mild	Mottling does not exceed the middle thigh
4	Severe	Mottling does not exceed beyond the fold of the groin
5	Grave	Mottling exceeds beyond the fold of the groin

Adapted from Misango *et al.*[38] – open access paper.

### Capillary refill time

Targeting a capillary refill time 3 s or less was shown as an adequate endpoint for fluid resuscitation [9]. One multicenter randomized clinical trial in 28 ICUs in five countries (Argentina, Chile, Colombia, Ecuador, Uruguay) in patients with septic shock showed that a strategy using normalization of capillary refill time as a resuscitation target was associated with a lower 28-day mortality, and faster resolution of organ dysfunction when compared with a strategy targeting normalization of serum lactate concentrations [10^▪^]. Several factors, however, may affect the accuracy of capillary refill time, including the temperature and light intensity in the room, the site of measurement and the amount of pressure applied to the capillary bed. Related to this, there have been concerns about the high interobserver variability in assessing capillary refill time [11].

### Skin temperature gradient

Skin temperature gradients, defined as the difference in skin temperature between an extremity and a more proximal skin zone, for example, between the fingertip and forearm, or between the toe and central core [12], can be used to identify shock, and is especially used in septic children, where hypotension is often a late phenomenon in the development of septic shock. The advantage of using skin temperature gradients between, for example, the fingertip and the forearm, instead of a single location, is that both spots will be similarly affected by ambient temperature. The normal skin temperature gradient between the fingertip and the forearm is 0 °C. Skin temperature gradients above 4 °C are associated with peripheral hypoperfusion. A normal or increased skin temperature gradient is correlated with improved survival in patients with sepsis [13]. However, a large trial in African children with severe febrile illness and compensated shock, mainly defined by a decreased temperature gradient in the absence of hypotension, showed that fluid bolus therapy with 20--40 ml/kg increased mortality compared with more conservative fluid management [14].

## LABORATORY ASSESSMENTS

### Blood lactate

Monitoring blood lactate can help identify patients at risk for adverse outcomes in the emergency department in LMICs [15^▪^]. Additionally, the reduction of lactate concentration is an established endpoint for resuscitation in critically ill patients [16]. Currently, point-of-care devices are available for rapid and inexpensive assessment of blood or plasma lactate concentrations, which can be a valuable asset in LMICs [17]. Blood lactate has a strong prognostic significance for case fatality in LMICs [15^▪^], including in tropical diseases like severe malaria [18].

## MICROCIRCULATORY MONITORING

### Orthogonal polarization spectral imaging and sidestream darkfield imaging

Microcirculatory monitoring techniques enable direct visualization of capillary blood flow with a microscopic camera, which can be placed on the sublingual or rectal capillary beds. Specific software has been developed to quantify capillary perfusion. Although some groups have advocated the use of sublingual capillary perfusion assessed by these devices as a cardiovascular resuscitation endpoint [19], this has not yet been adapted widely. In addition, the devices are produced only at a small scale, are relatively expensive, require training before they can be implemented, and therefore, less feasible for use in LMICs.

## CARDIOVASCULAR MONITORING

### Clinical assessments and basic noninvasive monitoring

Frequent basic physical examination using simple and cheap tools provide crucial information on the hemodynamic status of the critically ill patient. This includes automated blood pressure measurements, basic three-lead electrocardiography to monitor heart rate and rhythm, and monitoring of the pulse contour. A mean arterial blood pressure (MAP) of at least 65 mmHg is usually considered adequate. However, in the individual patient, tissue perfusion can be highly inadequate despite an appropriate blood pressure, for instance because of severe vasoconstriction. Conversely, some patients tolerate a low blood pressure very well, without any sign of tissue hypoperfusion, including the brain. Thus, interpretation of the blood pressure values always needs to be personalized.

The pulse pressure is the difference between the SBP and DBP. In patients with severe dengue, characterized by a generalized capillary leak, the pulse pressure is recommended for guiding fluid therapy [20]. A narrow pulse pressure is associated with a low cardiac output, for example, during hypovolemic or cardiogenic shock, whereas a wide pulse pressure is associated with a high cardiac output, for example, during septic or anaphylactic shock.

A weakening of the pulse during inspiration of more than 10 mmHg in SBP is called a pulsus paradoxus and can be caused by a cardiac tamponade, constrictive or restrictive pericarditis, or severe bronchial asthma.

### Echocardiography

Echocardiography is increasingly available in LMICs, and offers direct bedside assessments of stroke volume (SV), cardiac output, and valvular disease. Unlike echocardiography, traditional cardiac output assessments using intravascular catheters and dilution techniques are inaccurate in the presence of right heart failure, several valvular lesions, and arrhythmias. Echocardiography is easy to integrate with other point-of-care ultrasound techniques, such as lung or abdominal ultrasound, and is also useful for differentiating types of shock [21^▪^]. However, these ultrasound assessments require an experienced and skilled operator.

The left ventricle is assessed by echocardiography for end-diastolic diameter, shape, and gross abnormalities of contractility. The right ventricle is assessed for size and shape relative to the left ventricle, position of the interventricular septum, and free wall longitudinal contractility [22]. In the context of acute cardiovascular collapse, gross right ventricle impairment can be an indicator of right ventricle ischemia or infarction, as well as increased right ventricle afterload, for instance, caused by high levels of intrathoracic pressure, or pulmonary vascular resistance caused by lung emboli. In patients suspected of lung emboli, Doppler ultrasound can be used for checking deep vein thrombosis [23]. Echocardiography can also identify pericardial effusion, and rapidly assess left ventricle and valve function in patients with cardiogenic shock [21^▪^].

### Noninvasive or minimally invasive cardiac output monitoring

Noninvasive or minimally invasive methods to estimate cardiac output use noninvasive devices with specific algorithms, for example, noninvasive pulse contour analysis, thoracic bioreactance, Doppler ultrasound cardiac output monitoring, or minimally invasive pulse contour analysis, which require a peripheral arterial line. However, the accuracy of noninvasive cardiac output estimation is generally inferior to methods using thermodilution [24], require callidabration, and the reliability of minimally invasive pulse contour monitoring of cardiac output and derived variables is critically dependent on the quality of the arterial pressure signal. This usually limits its use in LMICs, because of unavailability of arterial pressure-measuring systems, including pressure transducer or flush system. In addition, use of arterial lines in resource-limited settings can have important safety concerns, including risks of infection, bleeding and arterial thrombosis.

### Invasive cardiac output monitoring

Monitoring through invasive catheters, including central venous catheters, pulmonary artery catheters, and arterial catheterization for invasive techniques including Fick's method or thermodilution have limited applicability in resource-limited settings, because of complexity of the procedure, costs, and other factors. Moreover, independent of the setting, the use of pulmonary artery catheters was shown to have no effect on important outcomes in intensive care patients [25].

## MONITORING FLUID STATUS AND PREDICTING FLUID RESPONSIVENESS

### Clinical assessment and basic tools

Clinical assessment of the jugular venous pressure is an insensitive measure of intravascular volume status, as well as fluid responsiveness [26]. Lung auscultation for the detection of crackles can identify increased extravascular lung water and pulmonary edema but less reliable in mechanically ventilated patients [27]. In the abdomen, significant fluid overload manifests as ascites or presence of a hepatojugular reflux. In the extremities (primarily the legs), fluid overload can cause limb edema.

A central venous jugular or subclavian line is part of standard care in most ICUs, also in LMICs. This allows for quick assessment of the central venous pressure (CVP) representing the filling pressure of the right side of the heart, which can be used as a measure of the intravascular filling status. However, a systematic review showed a poor correlation between the CVP and the response to fluids [28].

### Additional tools to monitor fluid status

Fluid responsiveness is defined as the ability of the heart to increase the cardiac output in response to volume expansion. Fluid responsiveness indicates the patient position on the Starling ventricular function curve, and can thus identify patients that are ‘over the top’ of the Starling curve who will not benefit from fluid loading. Although dynamic transthoracic echocardiogram measures are one of the reference standards for assessing fluid responsiveness in LMICs [29], there are several limitations to their use. These include that the method has been mainly validated in mechanically ventilated sedated patients without spontaneous respiratory effort, and with tidal volumes used for invasive ventilation above 7 ml/kg, with a normal intra-abdominal pressure, and an intact thorax wall [30].

Transthoracic echocardiography can be used to estimate the left ventricle filling pressure, which can guide fluid management [31^▪^]. Systolic obliteration of the left ventricle cavity or ‘kissing’ of the walls may be a sign of severe hypovolemia, although it can also be present in a number of other conditions. Left ventricular end diastolic pressure as a static marker has limited predictive value for fluid-responsiveness but can help to diagnose and guide management of cardiogenic pulmonary edema [30]. The right atrial pressure can be estimated from assessment of the inferior vena cava diameter in expiration and response to an inspiratory sniff. Right atrial pressure cannot be predicted accurately in the patient with several respiratory conditions, right ventricle failure, and increased intra-abdominal pressure, which all affect inferior vena cava-derived indices [32].

In invasively ventilated patients receiving tidal volumes at least 8 ml/kg predicted body weight and positive end-expiratory pressure (PEEP) 5 cmH_2_O or less, the inferior vena cava distensibility index, defined as (maximum inferior vena cava − minimum inferior vena cava)/minimum inferior vena cava × 100 (%) [33], is an accurate predictor of fluid responsiveness, with overall an excellent sensitivity and specificity. However, in patients who receive ventilation with a tidal volume less than 8 ml/kg predicted body weight, or PEEP above 5 cmH_2_O, the inferior vena cava distensibility index is inaccurate for predicting fluid responsiveness [34]. Also in spontaneous breathing patients, the inferior vena cava collapsibility index, defined as (maximum inferior vena cava − minimum inferior vena cava)/maximum inferior vena cava × 100 (%), is an inaccurate predictor of fluid responsiveness. Intensivists should be cautious when using these under such conditions [34].

Mini-fluid challenge test can help predict fluid responsiveness in patient receiving invasive ventilation [35]. Transthoracic echocardiography could be a reliable alternative to assess changes in SV or cardiac output [36,37].

The passive leg raising test is a feasible and affordable tool to guide fluid resuscitation in patients with or without invasive mechanical ventilation, including in resource-limited settings [38]. It is currently still uncertain whether the passive leg raising test has predictive values in all types of shock. Transthoracic echocardiography measurement of changes in cardiac output when performing a passive leg raising test is reliable [39]; using changes in pulse pressure [40] or capillary refill time [41] as alternative read-outs ultrasound is not available, has also shown good performance. Some conditions, however, including abdominal or intracranial hypertension and traumatic hip or lower limb fractures, limit the use of passive leg raising [39].

In patients receiving invasive ventilation, the intrathoracic pressure increases during the inspiratory phase resulting in a decrease of venous return. In the end-expiratory occlusion test, the increase in intra-thoracic pressure is temporarily prevented, causing an increase in venous return, cardiac preload, and SV in preload-responsive patients. Therefore, an increase in cardiac index during the end-expiratory occlusion test can predict the fluid responsiveness [35]. In order to identify the rapid and transient increase in cardiac index during an end-expiratory occlusion test, transthoracic echocardiography is used to assess the output velocity--time integral [42].

## EXTRAVASCULAR LUNG WATER MONITORING

Extravascular lung water is a key variable in fluid resuscitation. Ultrasound provides an easy and reliable method to estimate extravascular lung water. An increase of extravascular lung water creates so called B-lines, which are comet-like signals generated from hyperechoic subpleural interstitial edema [43^▪^]. The normal reflection pattern creates A-lines in the healthy lung, whereas increasing extravascular lung water creates single to multiple B-lines, accumulating into a ‘white’ lung caused by coalescing B-lines in patients with overt pulmonary edema [44].

Estimation of extravascular lung water through transpulmonary thermodilution devices, like the Pulse index Continuous Cardiac Output, is in general less suitable for ICU settings in LMICs as these require expensive invasive catheters, pressure transducers, and monitoring devices [45].

## CONCLUSION AND RECOMMENDATIONS

**FIGURE 2:**
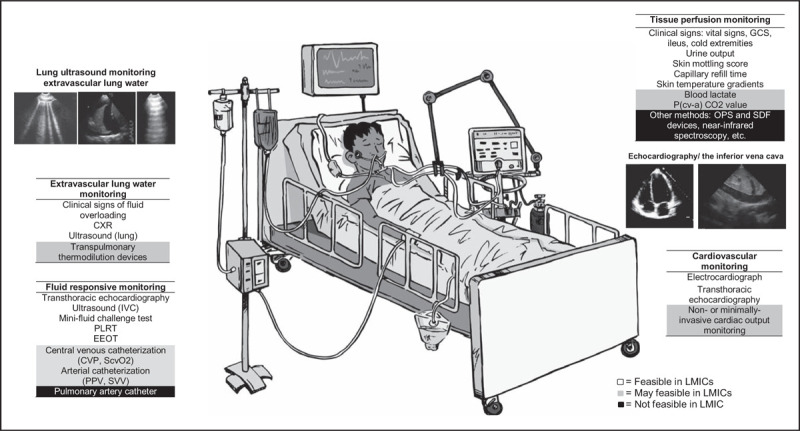
Available, affordable and feasible monitoring tools for use in low-income and middle-income countries. CVP, central venous pressure; CXR, chest x-ray; EEOT, end-expiratory occlusion test; GCS, Glasgow Coma Scale; IVC, inferior vena cava; OPS, orthogonal polarization spectral; P(cv-a) CO2, central venous-to-arterial carbon dioxide difference; PLRT, passive leg raising test; PPV, pulse pressure variation; ScvO2, central venous oxygen saturation; SDF, side stream dark-field; SVV, stroke volume variation.

Approaches and tools for hemodynamic monitoring in LMICs will have to be operated often in settings with challenging environmental conditions, a complex supply chain, inadequate operator training, and limited resources for purchasing and maintaining equipment. At the same time, the approaches and tools will need to have comparable performance and reliability as those for use in resource-rich settings. Hemodynamic monitoring that uses invasive, complicated procedures or expensive devices will often not be available, affordable or feasible in LMICs. Ultrasonography skills for hemodynamic monitoring using low-cost, portable ultrasound devices can be easily acquired by a variety of medical personnel, and monitoring by ultrasound techniques is recommended for ICUs in LMICs. Setting-appropriate cardiovascular monitoring approaches are summarized in Fig. 2.

## Acknowledgements

*We thank Professor Kunchit Piyavechviratana for his support and advice.*

### Financial support and sponsorship

*Professor Arjen M. Dondorp is supported by the Wellcome Trust of Great Britain (220211/Z/20/Z).*

### Conflicts of interest

*There are no conflicts of interest.*

## REFERENCES AND RECOMMENDED READING

Papers of particular interest, published within the annual period of review, have been highlighted as:

▪ of special interest
▪▪ of outstanding interest
